# Translation of Korean Medicine Use to ICD-Codes Using National Health Insurance Service-National Sample Cohort

**DOI:** 10.1155/2016/8160838

**Published:** 2016-03-16

**Authors:** Ye-Seul Lee, Ye-Rin Lee, Younbyoung Chae, So-Youn Park, In-Hwan Oh, Bo-Hyoung Jang

**Affiliations:** ^1^Acupuncture and Meridian Science Research Center, College of Korean Medicine, Kyung Hee University, Seoul 130-701, Republic of Korea; ^2^Department of Medicine, Graduate School, Kyung Hee University, Seoul 130-701, Republic of Korea; ^3^Department of Medical Education and Medical Humanities, College of Medicine, Kyung Hee University, Seoul 130-701, Republic of Korea; ^4^Department of Preventive Medicine, College of Medicine, Kyung Hee University, Seoul 130-701, Republic of Korea; ^5^Department of Preventive Medicine, College of Korean Medicine, Kyung Hee University, Seoul 130-701, Republic of Korea

## Abstract

*Background*. Korean medicine was incorporated into the Korean Classification of Diseases (KCD) 6 through the development of U codes (U20–U99). Studies of the burden of disease have used summary measures such as disability-adjusted life years. Although Korean medicine is included in the official health care system, studies of the burden of disease that include Korean medicine are lacking.* Methods*. A data-based approach was used with National Health Insurance Service-National Sample Cohort data for the year 2012. U code diagnoses for patients covered by National Health Insurance were collected. Using the main disease and subdisease codes, the proportion of U codes was redistributed into the related KCD 6 codes and visualized. U code and KCD code relevance was appraised prior to the analysis by consultation with medical professionals and from the beta draft version of the International Classification of Diseases-11 traditional medicine chapter.* Results*. This approach enabled redistribution of U codes into KCD 6 codes. Musculoskeletal diseases had the greatest increase in the burden of disease through this approach.* Conclusion*. This study provides a possible method of incorporating Korean medicine into burden of disease analyses through a data-based approach. Further studies should analyze potential yearly differences.

## 1. Introduction

Efforts towards standardization and globalization of heath care are occurring in different aspects of medicine and health policy [1]. Traditional medicine is included in this work; since the founding of the Division of Traditional Medicine in the World Health Organization in 1972, traditional medicine, based on the International Classification of Traditional Medicine (ICTM), is being included in the current updates to the International Statistical Classification of Diseases and Related Health Problems (ICD), currently in its 10th revised edition and in the progress of being updated to the 11th edition [2]. The Korean Classification of Diseases (KCD) also reflects these efforts. In 2010, the third edition of the Korean Classification of Diseases of Oriental Medicine (KCDOM3) was incorporated into the Korean modification of the ICD-10, or KCD 6, using U codes (U20–U99) [3]. In this aspect, KCD 6 was groundbreaking as the first publication in which Western medicine and traditional medicine shared a common platform.

U codes (U00–U99), also called codes for special purposes, are in Chapter XXII of the fourth edition of ICD-10 [4]. While this chapter includes codes such as U04 for severe acute respiratory syndrome (SARS), most of the codes in this chapter were developed to incorporate patterns or disorders diagnosed through Korean medicine (U20–U99). In Korea, doctors of Korean medicine are advised to use KCD 6, which is based on Western medicine, as their primary code system; however, when the doctor cannot correlate the diagnosis specifically to KCD 6, the doctors are to supplement the diagnosis with a U code [5]. While KCDOM2 (1994), which was based on Korean medicine, was used by doctors of Korean medicine instead of KCD 5, the overlap and mismatch of some diseases between KCD and KCDOM caused serious confusion. Therefore, U codes were developed to support the patterns and symptoms diagnosed only through Korean medicine while incorporating many of the disease codes from KCDOM2 that showed similar characteristics to KCD 6 codes. For example, terminology in Korean medicine that refers to cancer was absorbed into KCD 6 because the symptoms of the two different codes were almost identical; however, terminology in Korean medicine referring to patterns of disorders, such as qi deficiency pattern/syndrome, remained under U codes [3]. Therefore, through the third revision, KCDOM eliminated the overlapping disease classifications between the previous KCDOM and KCD5 and reorganized the remaining disorders and patterns into U codes, which reduced possible duplicate coding and allowed pattern identification and diagnosis through Korean medicine. The incorporation of KCDOM3 into KCD 6 was also conducted to meet the needs of doctors of Korean medicine to more effectively reflect the patient's condition. As a result, one of the major characteristics of KCDOM3 is its relationship with KCD 6 [5].

One approach towards better health care is quantification of the burden of disease [6]. Burden of disease is a crucial input into health policy, because it provides an account of health loss due to different risks through a disease-by-disease analysis [7]. Most health analyses concentrate on mortality, thereby omitting nonfatal, chronic diseases that affect quality of life [8]. At the same time, a focus on noncommunicable or chronic diseases has gained support as the morbidity and comorbidity of chronic diseases in the general population have increased [9]. The measurement of the burden of diseases, or Global Burden of Disease Study (GBD), was initiated in 1992, with three major goals: (1) to provide information on nonfatal health outcomes, as most of the health policies are generally focused on mortality; (2) to develop epidemiological assessments for major disorders without bias; and (3) to quantify the burden of disease with a measure that could also be used for cost-effectiveness analysis [10]. Research is currently being conducted in different countries for diverse risk factors, such as recent analyses of the global burden of disease due to ischemic heart disease, and to determine if there is epidemiological convergence across countries [1, 11]. Different approaches have been taken in burden of disease studies, including disability weights to cover the burden of disease more elaborately [12]. The foremost milestone, one of the most important milestones, of the GBD study was the development of the composite indicator disability-adjusted life years (DALYs), which is being used throughout diverse academic research as a summary measure of the overall burden of disease and is expressed as the number of years lost due to ill-health, disability, or early death [8].

Using DALYs, the burden of different diseases and risk factors have been analyzed in Korea using nationally representative data provided by health-related government agencies such as the Health Insurance Review & Assessment Service (HIRA) and National Health Insurance Service (NHIS) [13]. To analyze the burden of disease using nationally representative big data, disease codes (KCD) that are collected as part of the patient's health care utilization have to be categorized by definitions of the causes of disability and death in previous burden of disease studies [14]. In other words, the disease codes are regrouped and redistributed into different clusters to define risk factors [15]. However, previous studies have not included the portion of health care utilization classified under U codes when calculating the burden of diseases by disability and death causes, although the nationally representative data include information for health care utilization coded under the U codes, such as the number of visits and costs [16]. Therefore, an understanding of the U codes from the perspective of Western medicine is needed to redistribute the uncalculated burden of diseases under U codes to other codes.

Because data with main disease codes not covered by KCD 6 were overlooked in previous studies of the burden of disease, this study hypothesized that the collection of the subdisease codes within a year of data collection would reflect what was covered by the main disease code. In other words, the assumption was that, within the annual collection of data, the combination of main disease code and subdisease code would cover the diseases for a patient throughout a year.

## 2. Materials and Methods

### 2.1. Structure of U Codes

U codes can be divided into three components (Table 1): Korean medicine disorders (U20–U33), Korean medicine patterns (U50–U79), and four constitution medicine patterns (U95–U98). Because the U codes were created to define disorders or patterns that could not be defined using the disease classification system of Western medicine presented in KCD 6, the disorders and patterns in the U codes do not correspond directly to disease names in the KCD. Therefore, to incorporate the U codes into the burden of disease algorithm of the KCD, the underlying disorders and diseases in Western medicine were analyzed in this study using a data-based approach, via a redistribution algorithm of U codes into KCD 6 codes.

### 2.2. Data Source

The National Health Insurance Service-National Sample Cohort (NHIS-NSC) of 2012, which includes data for 1 million patients, was used for data analysis. NHIS-NSC data provide information on the utilization of healthcare based on the NHI claims from medical institutions to the NHIS from inpatient and outpatient clinic visits for each individual patient [17]. NHI claims data contain principal and additional diagnoses, hospitalization/outpatient treatment, dates of examinations, medical fees, details of medical services, prescribed medications, hospital codes, and patients' sex and age and are categorized on the basis of the examination documented in the claims from the medical institutions [18]. For this study, the main disease and subdisease codes were collected for outpatients of Korean medicine clinics from the 2012 NHIS-NSC data. U codes as the main disease code were redistributed to the KCD 6 codes.

### 2.3. Data Analysis

#### 2.3.1. Redistribution of U Codes to KCD 6 Codes

The primary goal was to use the data from the U code visits that also had subdisease codes in 2012 to the remaining visits with only U codes as main disease codes and without any subdisease codes.

The method to redistribute the U codes to KCD 6 codes was derived from garbage codes [1]. A garbage code redistribution algorithm was developed in a previous study of the burden of disease to explain the unknown cause of death based on the underlying cause in ICD-10 [14]. Similarly, the redistribution algorithm of U codes to KCD 6 codes aimed to explain disorders or patterns not explained by Western medicine based on the underlying cause found in the KCD 6.

First, U codes as the main disease code were collected, which accounted for 151,967 visits in 2012. These data became the target for data analysis, which was conducted with the 30 most commonly used U codes, covering approximately 80% of the total U code visits. Then, the subdisease codes and their frequencies were collected. In this process, subdiseases coded with U codes, S codes (injury, poisoning, and certain other consequences of external causes), R codes (symptoms, signs, and abnormal clinical and laboratory findings, NEC), and Z codes (factors influencing health status and contact with health services) were excluded before determining the frequencies.

Before the redistribution of the main disease U codes to subdisease KCD 6 codes, a reorganizing process was conducted to rule out the codes that were irrelevant to the main disease codes. Subdisease codes can be used for diseases other than the main disease in many cases. To avoid this problem, only subdisease codes that were relevant to the main U codes were selected by doctors of Korean medicine, and the final decision was based on agreement of trained KMD doctors. For example, in the case of U303 (neck stiffness), the codes that were not directly related to pain or abnormal sensation of the neck, such as digestive disorders or urinary disorders, were removed. This process was based on consultation with medical professionals and professors and researchers at the College of Korean Medicine, Kyung Hee University, as well as review of the beta version of ICD-11, which includes traditional medicine in its structure based on the ICTM. By reviewing the beta version of ICD-11, the definition and explanation for each of the disorders or patterns in the U code were studied for the specific symptoms or signs replaced by KCD 6 codes. Symptoms or signs of the disorders or patterns in the U code that were not mentioned in the corresponding ICD-11 definition were removed before data analysis.

#### 2.3.2. Calculation of the Proportion of U Codes in KCD 6 Codes

After selecting the KCD 6 codes among the subdisease codes and calculating the frequencies, each of the frequencies was replaced with the ratio of each U code and KCD 6 code within the total frequency of the corresponding U code. For example, in the case of U303 (neck stiffness), the frequency of the KCD 6 code in the subdisease code was converted into an intercode proportion, which equaled 1, within U303:

(1) Inter-U  code  proportion=Frequency  of  KCD  6  sub  disease  codeTotal  frequency  of  the  corresponding  U  code.

Then, each of these proportions was expanded and converted into the proportion within the total 151,967 visits that was only coded by U code and therefore missed in the original analysis of burden of disease, comprising the target data for analysis:

(2) U  code-KCD  6  expected  frequency=Inter-U  code  proportion∗151,967.

Finally, this proportion within the missed data was converted into a proportion within the total frequency of corresponding KCD 6 codes in the year 2012. Through this process, this study was able to quantify the proportion of the burden of disease in each KCD 6 code that was related to a U code or how much the missed data coded by U code added to the proportion of each burden of disease based on the KCD 6 codes. This process was conducted for each of the KCD codes in the subdisease codes in the U code data:

(3) U  code  Proportion=U  code-KCD  6  expected  frequencyTotal  frequency  of  the  corresponding  KCD  6  code.

However, when the frequency of the corresponding KCD 6 codes did not exceed 1,500, which was about 1% of the total U code frequency in our data, this process could result in overfitting of the total data. The process was designed under the assumption that the diseases in the KCD 6 codes followed a normal distribution; however, when the morbidity of the disease is too low, this process could stretch the proportion over the actual morbidity. Therefore, in such cases, the actual frequency, instead of the expected frequency, within the total U code data was used to calculate the proportion

(4) U  code  Proportion=Frequency  of  KCD  6  sub  disease  codeTotal  frequency  of  the  corresponding  KCD  6  code

if the total frequency of the corresponding KCD 6 code was <1,500.

Furthermore, the cooccurrence of U codes and the corresponding KCD codes was visualized to show the relationship between the burdens of disease based on U codes and KCD 6 codes. Specifically, each of the inter-U code proportions was visualized to show the relationship between the U codes and KCD 6 codes in the NHIS-NSC data from 2012.

The data were analyzed using SAS 9.3 (SAS Institute), and the data were visualized using Python.

## 3. Results

### 3.1. Redistribution of U Codes, or Codes for Special Purposes, into KCD 6 Codes

Table 2 shows the 30 most commonly used U codes from the data in the NHIS cohort data from 2012 that also had subdisease codes and the number of U code visits in 2012 (*n* = 24,164). The remaining 151,967 visits had only U codes as the main disease codes, without any subdisease codes.

The most commonly reported U code was U303, or neck stiffness. The most commonly used KCD 6 code in this analysis was related to musculoskeletal diseases (M codes), followed by diseases of the nervous system (G codes). Diseases of the digestive system (K codes) and mental and behavioral disorders (F codes) were also common. For example, U303 (neck stiffness) was redistributed to the following KCD codes: M791 (myalgia), M626 (muscle strain), M759 (shoulder lesion, unspecified), M758 (other shoulder lesions), M750 (adhesive capsulitis of shoulder or frozen shoulder), M255 (pain in joint), M542 (cervicalgia), M548 (other dorsalgia), M751 (rotator cuff or supraspinatus tear of rupture [complete, incomplete] not specified as traumatic), M796 (pain in limb), M549 (backache NOS), M531 (cervicobrachial syndrome), M501 (cervical disc disorder with radiculopathy), M797 (fibromyalgia), M624 (contracture of muscle), G568 (interdigital neuroma of upper limb), and G439 (migraine, unspecified).

Because KCD 6 codes corresponding to U codes were reviewed using the beta version of ICD-11 and with medical professionals prior to the redistribution process, there were no KCD codes without any relevance to the corresponding U codes. The proportions of the U codes to the KCD codes were fairly evenly distributed following the redistribution to enable comparison of the data from the 24,164 visits to the remaining 151,967 visits with only U codes as the main disease codes and without any subdisease codes and the additional adjustments to prevent overfitting values in the redistribution table. The U code proportions ranged from <1% to approximately 20% of the burden of disease for each KCD 6 code; there were few high proportions for each of the U codes (Table 3).

### 3.2. Visualization of the Relationships between U Codes and KCD 6 Codes

**Figure 1:**
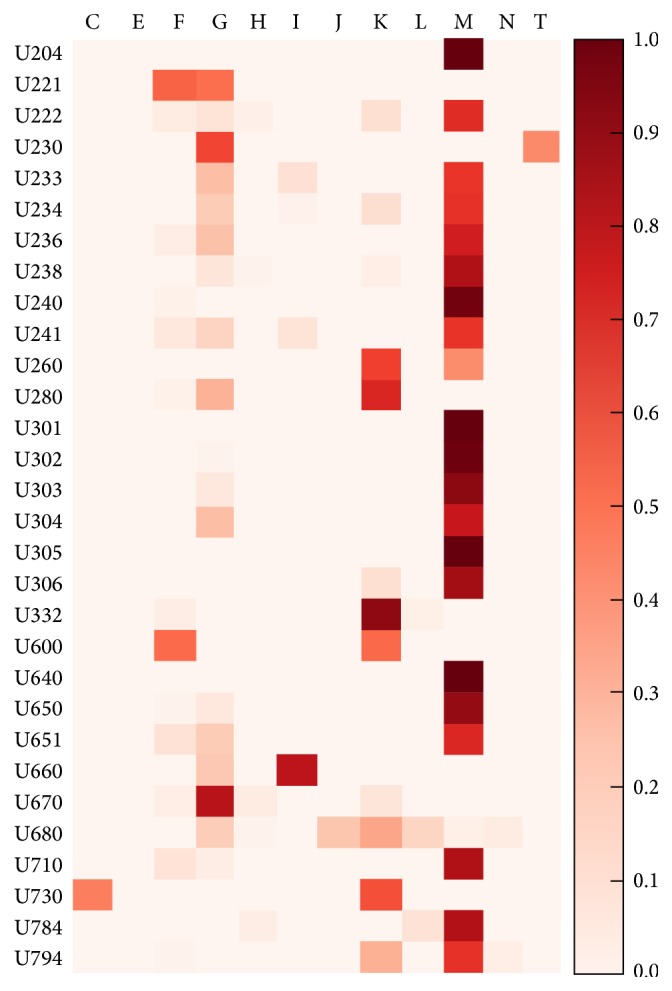
Visualization of the probability of the cooccurrence of the 30 most commonly used U codes in the 2012 National Health Insurance Service data in Korea and each chapter of the Korean Classification of Diseases 6 codes.

Figure 1 shows the data visualization of the 1-digit KCD 6 code in each U code, showing which KCD 6 chapter or disorder explains each U code and its proportion. A clear relationship between the 30 most commonly used U codes and musculoskeletal diseases is prominent. U codes that did not show a relationship with musculoskeletal diseases were U280 (food accumulation), U332 (night crying), U600 (qi deficiency pattern/syndrome), U670 (pattern/syndrome of heart fire flaming upward), U680 (pattern/syndrome of spleen qi deficiency), and U730 (pattern/syndrome of stomach qi deficiency). In contrast, these codes showed strong relationships with diseases of the nervous system (G codes) and diseases of the digestive system (K codes). There were two major U codes that had strong relationships with mental disorders (F codes): U600 (qi deficiency pattern/syndrome) and U221 (depression; melancholy; depressive syndrome). It is interesting to note that U222 (fire disease, hwa-byung), which is listed in the Diagnostic and Statistical Manual, Fourth Edition (DSM-IV), as a culture-bound syndrome, did not show a strong relationship with mental disorders but rather showed a clearly strong relationship with musculoskeletal diseases. The DSM-IV criteria indicate that hwa-byung has strong psychosomatic symptoms rather than direct mental symptoms [19].

## 4. Discussion

To our knowledge, this is the first study to incorporate U codes into the calculation of the burden of disease in Korea, with a specific focus on the analytic methods and results to assess the burden of diseases coded under U codes that have been overlooked in previous studies. Many of the U codes were redistributed within KCD 6 classifications for musculoskeletal diseases and diseases of the nervous system.

Until now, standardized compilations of methods for the analysis of traditional medicine in studies of the burden of disease have been lacking. Of the few studies that have focused on systematically understanding disease patterns explained in traditional medicine, some have shown possible links between the disorders and patterns and KCD or ICD [2, 20]. The present study, which enabled quantification of the utilization of health care services within Korean medicine, showed the additional proportion of the burden of disease for each KCD 6 code that could be assumed as the underlying factor in each of the U codes analyzed. Using this method, this study enabled a more complete analysis of the burden of disease in Korea, by including the part of the NHIS-NSC data represented by Korean medicine health care utilization. Information in the NHIS-NSC is organized by the type of medical institutions—Western medicine, Korean medicine, dental medicine, or pharmaceutical. NHIS provides an annual report, called the* National Health Insurance Statistical Yearbook*, which includes summaries of the utilization of each type of medicine from the NHIS-NSC data.  Table 4 provides the recent (2010–2012) trend in health care utilization by the type of medicine from the yearbook [21]; the utilization of Western medicine and Korean medicine did not drastically change over the years.

The redistribution of many of the U codes into musculoskeletal diseases and diseases of the nervous system based on the KCD 6 supports the results of previous studies, in which Korean medicine was mainly utilized for musculoskeletal diseases [22, 23]. These results reflect the current utilization of Korean medicine in health care; many of the patients who visit Korean medical clinics have these diseases. Approximately 30% of patients with musculoskeletal diseases visit Korean medical clinics for treatments such as acupuncture [24]. In addition, many patients with diseases of the nervous system, such as facial palsy, cerebral infarct, or dementia, visit traditional medicine hospitals [25, 26]. The present results, including those illustrated in Figure 1, should be understood within the current Korean medicine healthcare utilization, as part of the official health care system.

Although the data were limited to claims records from the NHIS-NSC, the results of the present study show how each of the disorders or patterns in Korean medicine can be understood in terms of KCD 6 codes. This data-driven approach provides a new perspective in understanding and explaining disorders and patterns in Korean medicine, or within the larger scope of traditional medicine, via the disease classification system in Western medicine [27]. Previous efforts have focused on academic or experimental approaches, providing explanations of the physiological or functional symptoms explained in Korean medical or traditional medicine texts through the scientific lens of Western medicine or biomedicine or suggesting a possible mechanism for disorders and patterns in Korean medicine through experimental methods [2, 20, 28]. In contrast, a data-driven approach does not rely on the prior categorization of diseases as latent variables; rather, the data-driven approach enables a direct comparison of diseases between Western medicine and Korean medicine through data.

There are a few limitations in our study. First, the data source was based on claims from medical institutions to the NHIS. In other words, the data source and analysis did not include health care services not covered by the NHIS, including the out-of-pocket (OOP) sector. It is important to note that the portion of Korean medicine health care service that is not covered by NHIS is fairly large; therefore, a large part of Korean medicine health care utilization would not have been reported in the NHIS-NSC data [29, 30]. Second, the analysis was conducted for the 30 most common U codes in the NHIS data for the year 2012, which could have produced two issues. First, the most common U codes could change by year, with trends in health care utilization, which could therefore change the burden of disease. Also, the proportion that this study added to the current analysis of the burden of disease could change over time, yielding different data in another year. However, since this study aimed to produce the proportion in which the burden of disease for the year 2012 could develop, these two problems did not cause major errors in the current project. Furthermore, we aim to continue this project and apply the same method to another year to see the possible changes in the assimilated U codes and their proportions.

## 5. Conclusions

This study analyzed the burden of disease from U codes in the year 2012 using NHIS-NSC data. Although there are some limitations, quantification of the proportion of U codes to KCD 6 codes and redistribution of those codes enable a better understanding of Korean medicine health care utilization. Furthermore, the relationship between U codes and KCD 6 codes through data visualization provides a way of understanding U code disorders and patterns from the KCD 6 perspective. Furthermore, it provided a deeper understanding of the disorders and patterns of U codes through KCD 6 diseases. This data visualization showed that musculoskeletal diseases accounted for a large part of Korean medicine utilization. Furthermore, the methodology applied in this study serves as an initial study to quantify U codes through KCD 6 codes, providing guidelines for further research of the burden of diseases, including other countries with a dual health care system similar to that in Korea.

## Figures and Tables

**Table 1:** Summary of U codes or code for special purposes in the Korean Classification of Diseases 6, which was revised in 2009.

3-digit code	Code name	Number of 4-digit subcategories
**U20–U33**	**Disease name in Oriental medicine**	**97**
U20-U21	General diseases	12
U22	Mental and behavioral disorders	3
U23-U24	Diseases of the nervous system	12
U25	Diseases of eye, tongue, and throat	6
U26	Diseases of the circulatory system	4
U27	Diseases of the respiratory system	8
U28	Diseases of the digestive system	10
U29	Diseases of the skin and subcutaneous tissue	8
U30	Diseases of the musculoskeletal system and connective tissue	7
U31	Diseases of the genitourinary system	10
U32	Diseases of the female genitourinary system and those related to pregnancy	8
U33	Diseases of retardation and development, childhood, and adolescence	9

**U50–U79**	**Disease pattern/syndrome in Oriental medicine**	**191**
U50	Disease pattern/syndrome of six excesses	9
U51–U57	Disease pattern/syndrome of the six meridians	45
	Greater yang disease pattern/syndrome	14
	Yang brightness disease pattern/syndrome	6
	Lesser yang disease pattern/syndrome	6
	Greater yin disease pattern/syndrome	3
	Lesser yin disease pattern/syndrome	10
	Reverting yin disease pattern/syndrome	6
U58	Disease pattern/syndrome of defense-qi-nutrient-blood	9
U59	Disease pattern/syndrome of triple energizer	4
U60–63	Disease pattern/syndrome of qi-blood-yin-yang-fluid-humor	30
	Disease pattern/syndrome of qi	6
	Disease pattern/syndrome of blood	6
	Disease pattern/syndrome of qi-blood-yin-yang	9
	Disease pattern/syndrome of fluid and humor	9
U64–U79	Disease pattern/syndrome of viscera and bowels	94
	Liver disease pattern/syndrome	14
	Heart disease pattern/syndrome	12
	Spleen disease pattern/syndrome	7
	Lung disease pattern/syndrome	11
	Kidney disease pattern/syndrome	8
	Gallbladder disease pattern/syndrome	4
	Stomach disease pattern/syndrome	5
	Large intestine disease pattern/syndrome	4
	Small intestine disease pattern/syndrome	3
	Bladder disease pattern/syndrome	2
	Disease pattern/syndrome of thoroughfare, conception vessels, and uterus	7
	Combined disease pattern/syndrome of viscera and bowels	17

**U95–U98**	**Disease pattern/syndrome of Four-Constitutional Medicine**	**18**
U95	Soeumin disease pattern/syndrome	5
U96	Soyangin disease pattern/syndrome	5
U97	Taeumin disease pattern/syndrome	5
U98	Taeyangin disease pattern/syndrome	3

**Table 2:** Thirty most commonly used U codes in Korea, 2012.

Code	Name	Frequency (2012)
U303	Neck stiffness	6,552
U240	Numbness	3,142
U234	Sequela of wind stroke	2,680
U670	Pattern/syndrome of heart fire flaming upward	1,684
U238	Impediment disease	1,323
U280	Food accumulation	768
U680	Pattern/syndrome of spleen qi deficiency	717
U301	Painful impediment	551
U305	Crane-knee arthritis	488
U304	Joint-running wind	480
U241	Insensitivity	462
U651	Pattern/syndrome of liver qi depression	458
U306	Muscle cramp	411
U236	Tremor	392
U222	Fire disease, hwa-byung	362
U221	Depression, melancholy, and depressive syndrome	326
U302	Fixed impediment	325
U650	Pattern/syndrome of ascendant hyperactivity of liver yang	306
U730	Pattern/syndrome of stomach qi deficiency	291
U230	Head wind	270
U710	Pattern/syndrome of kidney qi deficiency	264
U660	Pattern/syndrome of heart qi deficiency	246
U640	Pattern/syndrome of liver blood deficiency	233
U233	Prodrome of wind stroke	231
U794	Pattern/syndrome of spleen and kidney yang deficiency	225
U784	Pattern/syndrome of liver and kidney yin deficiency	222
U332	Night crying	199
U260	Chest impediment	197
U600	Qi deficiency pattern/syndrome	186
U204	Consumptive disease	173

**Table 3:** Redistribution of U codes to Korean Classification of Diseases (KCD) 6 codes.

U code	U code proportion to each KCD 6 code														
U303	M791	M626	M759	M758	M750	M255	M542	G442	M548	M751	M796	G568	M549	G439	M531
	9.25%	9.56%	9.02%	17.62%	5.62%	4.73%	2.79%	16.91%	11.08%	14.29%	2.96%	21.85%	7.75%	10.13%	9.37%
	M501	M797	M624												
	20.54%	7.90%	9.89%												

U240	M179	M626	M759	M758	M541	M538	M175	M171	F453						
	11.58%	8.49%	5.17%	6.85%	77.35%	7.55%	20.38%	2.70%	10.58%						

U234	M545	G518	G510	M179	M796	K30	M791	M544	K580	M255	M171	M759	G819	M542	M752
	1.39%	33.08%	6.12%	2.19%	3.47%	2.32%	1.09%	1.44%	35.92%	2.36%	4.34%	1.81%	7.82%	0.67%	7.98%
	I693	K590	G430	M626	M139	G810	I691	M199	M509						
	4.31%	8.95%	11.94%	1.39%	6.41%	14.68%	6.99%	2.94%	4.22%						

U670	G470	G519	G64	G442	G439	K088	H819	F454	G909	G438					
	44.07%	25.68%	6.55%	14.16%	15.26%	43.28%	2.67%	1.33%	4.41%	9.11%					

U238	M545	M179	M791	M255	M796	M626	M750	K30	G470	M759	G510	G560	M543	M199	H911
	0.69%	1.49%	0.79%	1.51%	1.24%	0.72%	1.24%	0.64%	5.18%	0.53%	1.08%	10.20%	0.86%	3.79%	14.29%
	M653	M544	M542	M549	G562	M170	M171	F329	M501	M708					
	1.33%	0.14%	0.17%	0.92%	20.69%	0.73%	0.40%	7.50%	2.30%	0.44%					

U280	K30	G442	K318	G438	G430	G439	F982	K590							
	5.06%	6.78%	9.77%	11.10%	8.41%	2.57%	1.80%	3.91%							

U680	K30	J310	L80	L208	G442	G700	G470	M542	G439	H521	K522	N944	N959	G245	J300
	1.89%	6.53%	5.40%	11.96%	2.77%	17.54%	2.71%	0.25%	1.53%	1.49%	5.43%	1.67%	2.69%	1.36%	1.59%
	K121	N946													
	0.64%	3.72%													

U301	M545	M759	M544	M511	M791	M543	M626	M171	M255	M549	M238	M624	M758	M796	M179
	0.28%	0.99%	0.50%	2.70%	0.16%	0.68%	0.20%	0.56%	0.30%	1.09%	2.41%	2.45%	0.44%	0.22%	0.09%
	M750	M771	M109	M139	M753	M797	M170	M700							
	0.16%	0.43%	2.50%	1.23%	2.24%	0.73%	0.24%	1.67%							

U305	M796	M179	M791	M159	M255	M626	M722	M729							
	2.17%	1.09%	0.49%	1.46%	0.83%	0.26%	3.94%	0.71%							

U304	G560	M545	M758	M544	M171	M179	M255	M759	M750	M542	M104	M626	M791	M199	
	36.40%	0.15%	1.86%	0.35%	1.16%	0.29%	0.36%	0.30%	0.38%	0.18%	6.82%	0.09%	0.05%	0.97%	

U241	M626	G20	I693	M179	F453	M759	M531	M758	M480	M511					
	1.66%	2.06%	4.35%	0.42%	4.99%	0.49%	2.69%	0.66%	1.00%	0.51%					

U651	M626	M791	G430	M759	M545	M544	M758	F453	G440	M751	G442	M255	F454	F411	F410
	1.06%	0.36%	15.46%	0.45%	0.06%	0.19%	0.72%	2.91%	3.35%	1.30%	1.11%	0.22%	0.50%	3.44%	4.92%
	G438	M624	M750												
	1.05%	1.01%	0.11%												

U306	M545	M791	K30	M796	M255	M626	M239	M759	M179	M543	M771	G560	F480	M542	M170
	0.02%	0.04%	0.06%	0.07%	0.05%	0.02%	1.10%	0.02%	0.01%	0.03%	0.05%	0.22%	0.55%	0.01%	0.02%

U236	M179	G258	M159	M626	F459	G250									
	2.26%	10.78%	2.68%	0.32%	0.68%	0.54%									

U222	M545	M791	K30	G470	M759	M626	M255	F454	H571	F510	M199	M519	M542	M722	M796
	0.15%	0.39%	0.47%	3.89%	0.35%	0.15%	0.20%	0.75%	5.14%	4.85%	1.30%	0.96%	0.10%	1.21%	0.13%
	K219	M179	F453	G438	G442	I119									
	2.08%	0.04%	0.31%	0.40%	0.16%	3.66%									

U221	G470	F453	F519	G438	F454										
	16.34%	15.55%	6.88%	4.73%	0.41%										

U302	M179	M545	M791	M480	M624	M796	M626	M792	M544	M17	M508	M541	G430	G64	M139
	0.69%	0.13%	0.17%	2.77%	4.38%	0.41%	0.20%	3.76%	0.05%	4.09%	1.21%	1.67%	0.88%	0.32%	0.65%
	M235	M542													
	0.51%	0.03%													

U650	M545	M791	M626	M171	M544	G438	G439	G519	F502	M542	M796	M255	M549		
	0.21%	0.35%	0.35%	0.97%	0.14%	1.49%	0.60%	0.61%	8.57%	0.06%	0.08%	0.05%	0.21%		

U730	K30	C259	K295	K296	C169										
	1.46%	19.57%	6.81%	3.75%	1.23%										

U230	T676	G244	G442												
	11.43%	0.56%	5.82%												

U710	M478	M545	F453	M791	M626	G438	M179	M255	N951						
	5.22%	0.13%	3.95%	0.14%	0.17%	1.98%	0.07%	0.11%	2.29%						

U660	G700														
	8.77%														

U640	M253	M758	M759	M249											
	1.14%	2.32%	0.89%	0.29%											

U233	M622	M179	G442	I630	M796	G501	G819	G909							
	2.26%	0.57%	1.89%	15.00%	0.45%	0.42%	0.76%	0.55%							

U794	K30	M171	M791	M626	N318	M544	M179	F458	F480	M750	M758	M796	N944		
	0.76%	1.72%	0.26%	0.36%	17.14%	0.04%	0.04%	1.40%	0.33%	0.02%	0.04%	0.02%	0.56%		

U784	M543	M626	M545	L031	M242	M791	H111	M199	M489	M549	E282	M759			
	1.70%	0.36%	0.06%	2.15%	3.38%	0.09%	2.64%	1.30%	3.64%	0.48%	3.39%	0.04%			

U332	K30	F982	L211												
	2.10%	1.20%	6.67%												

U260	K219	M626	M624												
	13.19%	0.58%	3.33%												

U600	F500	K30	K590												
	5.17%	0.72%	6.42%												

U204	M545	M750	M759	M796	M170	M544	M626	M255	M772						
	0.09%	0.67%	0.36%	0.42%	0.85%	0.09%	0.08%	0.09%	0.94%						

Values are reported as the proportion of the U code in each KCD 6 code, %.

**Table 4:** Trend in health care utilization from the *National Health Insurance Statistical Yearbook*, Korea.

	2010	2011	2012
Number of patients			
WM	44,818,780 (77.9%)	45,200,513 (78.0%)	45,764,919 (78.1%)
KM	12,689,192 (22.1%)	12,724,688 (22.0%)	12,795,918 (21.9%)
Total treatment cost ($)			
WM	31,211,729,553 (94.8%)	33,173,091,418 (94.7%)	34,616,590,233 (94.6%)
KM	1,701,831,541 (5.2%)	1,838,759,399 (5.3%)	1,962,494,521 (5.4%)
Number of total claims			
WM	604,017,783 (79.0%)	615,979,142 (79.3%)	682,586,833 (80.3%)
KM	91,356,214 (12.0%)	92,010,198 (11.8%)	96,378,959 (11.3%)
Number of outpatient claims			
WM	593,702,030 (78.8%)	605,084,745 (79.0%)	670,812,474 (80.0%)
KM	91,227,649 (12.1%)	91,850,417 (12.0%)	96,181,670 (11.5%)

WM: Western medicine; KM: Korean medicine.
